# Dr. Hopestill R. Phillips

**Published:** 1916-09

**Authors:** 


**Dr. Hopestill R. Phillips** of Penn Yan (not listed in Polk nor State Directory) died July 19. He was a Confederate veteran. He leaves two sons in the profession, Wm. H. of Bath and Samuel A. P. Phillips of Coudersport.

